# Socioeconomic Status and Obesity

**DOI:** 10.1210/jendso/bvae176

**Published:** 2024-10-07

**Authors:** Kristen Autret, Traci A Bekelman

**Affiliations:** Lifecourse Epidemiology of Adiposity and Diabetes (LEAD) Center, Colorado School of Public Health, Aurora, CO 80045, USA; Lifecourse Epidemiology of Adiposity and Diabetes (LEAD) Center, Colorado School of Public Health, Aurora, CO 80045, USA

**Keywords:** social determinants of health, disparities, adiposity, mediator, moderator, research gaps

## Abstract

Obesity is a pervasive public health problem that causes debilitating complications across the life course. One opportunity for preventing the onset of obesity is to focus on its social determinants. Socioeconomic status (SES), which includes factors such as income, educational attainment, occupational prestige, and access to resources, is a key determinant of obesity. In this scoping mini-review, we summarized review articles and meta-analyses of the SES-obesity association. From the 1980s to the present, cross-sectional studies have demonstrated a persistent socioeconomic gradient in obesity in which the association is negative in developed countries and positive in developing countries. Longitudinal studies have revealed the bidirectionality of the SES-obesity association; some studies demonstrate that socioeconomic adversity precedes the onset of obesity, while others provide evidence of reverse causality. While earlier studies relied on anthropometric assessments of weight and height to define obesity, the use of modern technologies like dual-energy x-ray absorptiometry and bioelectrical impedance have demonstrated that the socioeconomic gradient in obesity is robust across multiple indicators of body composition, including direct measures of lean and fat mass. More recently, examination of mediators and moderators of the SES-obesity association have highlighted causal pathways and potential intervention targets, with a focus on health behaviors, environmental conditions, psychological factors, and biological processes. We describe current gaps in knowledge and propose opportunities for future innovation to reduce the burden of obesity and related socioeconomic disparities.

Rates of obesity and its associated conditions, such as type 2 diabetes, metabolic syndrome, and nonalcoholic fatty liver disease, are increasing globally [1-3]. Obesity predicts premature death and causes debilitating complications across the life course, including cardiovascular disease, certain types of cancers, higher rates of depression, and worse health-related quality of life [4, 5]. In addition to these health effects, the consequences can extend beyond the individual with obesity. The economic effect of obesity, both in health care expenditures and lost productivity, is high [6-8]. Furthermore, excess adiposity among adults can propagate disease in future generations via the intergenerational transmission to offspring [9, 10].

One opportunity for reducing the burden of obesity and preventing its onset is to focus on its social determinants [11, 12]. The social determinants of health (SDoH) have been described as the conditions in which people are born, grow, live, work, and age that affect their health [13]. This includes protective or harmful conditions that are often disproportionately allocated in the population in ways that systematically benefit or disadvantage some groups over others [14]. One prominent example of an SDoH in the health sciences literature is socioeconomic status (SES) because it is thought to be a key determinant of a wide range of health outcomes, including obesity, type 2 diabetes, and cardiovascular disease [15-20]. SES, broadly defined, encompasses income, educational attainment, occupational prestige, perceptions of social status, and access to opportunities and resources. Socioeconomic conditions can influence health via multiple pathways: health behaviors, access to health care, environmental exposures, physiological processes, cultural ideals of health, and psychological factors [21]. The ongoing and projected increases in income inequality, at least in some countries, may intensify the activation of these pathways, and exacerbate health inequities [22].

In a seminal review paper published in the 1980s on the topic of SES and obesity, Sobal and Stunkard summarized the direction of the SES-obesity association worldwide [23]. SES and obesity are positively associated in countries with low-income economies and inversely associated in countries with high-income economies [23]. Since then, decades of studies showed that these gradients have persisted, although most studies focused on cross-sectional associations among adult women [24-31]. Despite the persistence of this patterning, the factors that mediate and modify these associations likely have evolved over time due to major shifts in urbanization, globalization, technologies of convenience, global nutrition transitions, and cultural ideals about food, exercise, and body type [32-34]. Socioeconomic conditions are difficult to change, so understanding modifiable pathways and resilience factors that may buffer the effects of SES on obesity can help reduce health inequities. Furthermore, the methodologies to examine body fat composition and distribution have evolved over time, and early studies of SES and obesity that relied on anthropometric measures of weight and height have been repeated using more rigorous measures of adiposity that can distinguish between lean and fat mass, such as dual-energy x-ray absorptiometry, bioelectrical impedance, air-displacement plethysmography, and 3-dimensional body scanning [27, 35].

The purpose of this scoping review was to summarize previous work on the SES-obesity association from the last two decades and highlight potentially modifiable pathways and resilience factors that could stimulate the design of randomized controlled obesity-prevention trials. We focused on individual- and household-level SES and future opportunities to advance the science on obesity prevention among those in households with lower SES.

## Materials and Methods

### Search Strategy

To identify relevant studies examining the relationship between SES and obesity, we conducted a literature search from March to May 2024 using PubMed and EMBASE. The search strategy employed multiple combinations of the following search terms: “*socioeconomic status*,” “*income*,” “*education*,” “*overweight*,” “*obesity*,” “*adiposity*,” “*body mass index*,” “*body composition*,” and “*body fat distribution*” in the title or abstract. Articles were prioritized for review if they met the following criteria: (1) published in English, (2) published in the last 20 years (since 2004), (3) SES was the predictor variable and obesity or some other measure of adiposity was the outcome variable, (4) review articles or meta-analyses published in a peer-reviewed journal that followed PRISMA (Preferred Reporting Items for Systematic Reviews and Meta-Analyses), or similar, guidelines, and (5) multicountry papers, although we included country-specific reviews when they provided new insights. When no recent review papers were found, we searched for single-study original research articles. We excluded reviews of qualitative studies and experimental study designs. The “Results” section describes the findings from 19 review articles and meta-analyses published between 2007 and 2024 (Supplemental Table 1) [36]. Although a few reviews incorporated individual studies published prior to 2000, the majority of reviews included studies published after 2000, ensuring that the synthesis presented here is based primarily on recent data. We compared the bibliographies across all 19 articles to ensure that overlap was minimal and that we did not overemphasize the results from any given study.

The screening process was conducted in 2 stages. In the first stage, titles and abstracts of all identified studies were independently screened by 2 reviewers (K.A. and T.A.B.). In the second stage, the full texts of the selected articles were reviewed. Additionally, reference lists of the included studies were manually searched to identify any additional relevant articles missed during the initial database search.

### Definitions of Socioeconomic Status and Obesity

The American Psychological Association defines SES as encompassing not only income but also educational attainment, occupational prestige, and subjective perceptions of social status and social class. According to the American Psychological Association definition, SES also reflects quality-of-life attributes and opportunities afforded to people within society (eg, access to health care) [37]. This definition is further expanded to encompass material capital (eg, property), human capital (eg, skills, abilities), and social capital (eg, social networks, power) as a reflection of the status of individuals in the social structure [38]. While the construct of SES is holistic and complex, it is often operationalized in the health sciences literature using concrete, one-dimensional indicators such as annual household income or years of formal education. Thus, many of the papers included in this review used the variables income and educational attainment to operationalize SES.

The World Health Organization defines obesity as a complex chronic disease characterized by excessive fat accumulation that can impair health [39]. Among adults, the most commonly used indicator is body mass index (BMI), calculated as body weight in kilograms divided by height in meters squared (kg/m^2^). The cutoff for adult obesity is a BMI of 30 or greater [39]. Among children, multiple weight- and height-based indicators that are sex and age specific have been used to define obesity [40]. While BMI is correlated with total body fatness, BMI is limited in that it does not distinguish between lean and fat mass or describe how fat is distributed around the body [41, 42]. Another limitation of BMI is that the association between BMI and percentage of body fat can vary by age, sex, and ethnic group [43]. There are many indicators of fat mass, fat-free mass, and body fat distribution, but these measurements are used less frequently than BMI, possibly because they are relatively expensive and burdensome to collect compared to weight and height. Thus, the majority of papers included in this review used BMI to categorize individuals as having or not having obesity. Nevertheless, the search strategy included terms to capture studies that examined more rigorous measures of body composition and body fat distribution, given their association with disease risk independent of BMI [44].

## Results

### Cross-sectional Studies of Socioeconomic Status and Obesity

We evaluated 10 review articles published in the last 20 years focused on cross-sectional associations between SES and obesity [15, 24, 25, 31, 45-50]. The review articles collectively incorporated multiple indicators for obesity (eg, continuous BMI or BMI category) or SES (education, income, occupation, composite index, area-level deprivation, employment status, property ownership). The study samples included children, adolescents, and adults living in countries with low-, middle-, and high-income economies, and data were collected over 3 decades from the late 1980s to 2021. Findings were generally consistent across all 10 review articles. After stratifying the findings by national income, SES and obesity were positively associated in countries with low-income economies and inversely associated in countries with high-income economies. The SES-obesity association tended to be stronger among females compared to males. In countries with middle-income economies, the SES-obesity gradient was inconsistent across articles or flat, possibly because the positive association between SES and obesity was transitioning to an inverse one as countries were experiencing economic development [47].

### Longitudinal Studies of Socioeconomic Status and Obesity

The present review article conceptualized SES as the exposure variable. Cross-sectional studies have provided valuable insights, but their findings should be interpreted with caution because these study designs cannot establish a temporal relationship between SES and obesity. There are several plausible pathways by which obesity could lead to lower SES among some individuals. Examples include the possibility that individuals with obesity may enter into lower-income jobs due to labor-market discrimination or obesity contributing to high health-care expenditures that pull individuals below the poverty line, although this is certainly not the case for all individuals with obesity [51]. Longitudinal studies can establish temporality by following individuals over time and measuring SES and obesity at multiple time points. These types of studies can help identify windows of susceptibility and specific life stages (eg, in utero, early adulthood) when SES may have the greatest effect on obesity.

We identified 2 systematic reviews and meta-analyses that summarized the findings from longitudinal studies of the association between SES and obesity [51, 52]. All studies included in the reviews were conducted in high-income countries, although this was not an inclusion criterion. A 2017 meta-analysis by Newton and colleagues [52] (14 studies) focused on studies among adults that included life course SES as the exposure, operationalized with variables such as childhood family income or father's occupation. Among females, there was a consistent association between lower life course SES and higher BMI, obesity prevalence, and waist circumference. Among males, the pattern differed, with lower life course SES not associated with obesity but showing a positive association with waist circumference. A 2018 meta-analysis by Kim and von dem Knesebeck [51] focused on prospective studies of the association between income and obesity. Of the 21 studies included this review, 14 showed an inverse association between SES and subsequent obesity, while 7 provided evidence for reverse causality .

### Associations Between Socioeconomic Status and Direct Measures of Body Composition

Among adults and children, there is some evidence that the association between SES and obesity may vary depending on which indicator of adiposity is used [53, 54]. Specifically, the use of BMI may overestimate or underestimate the socioeconomic gradient in obesity compared with studies that examine fat distribution or body composition [53, 54]. Two systematic reviews published in 2021 examined the association between socioeconomic status (eg, income, education, overcrowding, area-level deprivation) and direct measures of body composition (eg, using dual-energy x-ray absorptiometry or bioelectrical impedance, and not anthropometric measurements of weight and height to calculate BMI) [27, 28]. Among adults and children in countries with high-income economies, there was an inverse association between SES and fat mass, especially among females. In countries with middle-income economies, the direction of the association was reversed, so that lower SES was associated with lower fat mass. Regardless of national income or life stage, higher SES was generally associated with greater lean mass [27, 28].

### Mediators of the Socioeconomic Status–Obesity Association

Two systematic reviews summarized studies examining mediators of the SES-obesity association among children in high-income countries [55, 56]. Both reviews identified evidence for a mediating role of television viewing, parent BMI, breastfeeding duration, breakfast omission, childcare attendance, and some dietary behaviors. Other potential mediators were explored (birth weight, physical activity, fruit and vegetable intake, smoking during pregnancy), but findings were inconsistent across studies, so the mediating role of these variables is inconclusive [55, 56]. Another systematic review of mediators among youth in Ireland and the United Kingdom confirmed many of these findings, and also identified evidence for the mediating role of access to green space and favorable neighborhood conditions [57]. Among adults, a 2019 systematic review of psychosocial pathways between SES and obesity in high- and middle-income countries indicated a mediating role for neighborhood quality, stress, self-esteem, expectations for the future, and early-life adversity (eg, low parental support), although the pathways differed by sex. For example, stress and social support were more important mediators among women compared to men. Job stress, social support, attitudes and motivation toward health behaviors, self-efficacy, and personality characteristics were examined as potential mediators in only a small number of studies, or findings were mixed; thus, the mediating role of these factors merits further study [58]. The aforementioned reviews focused on the mediating role of health behaviors and social/psychosocial factors. No reviews were identified for biological mediators of the SES-obesity association, but individual studies have highlighted areas where additional research may be warranted. This includes a mediating role for epigenetics (eg, DNA methylation) [59], inflammatory markers (eg, C-reactive protein) [60], markers of chronic stress [61], sex-specific biological factors (eg, sex hormones) [62], and immune system dysregulation and adipokine secretion [61], any of which could increase adipose tissue accumulation, central fat deposition, or insulin resistance [59-61, 63-65]. Testing hypotheses about biological mediation requires longitudinal studies that collect biospecimens and measures of SES and obesity at multiple time points.

### Moderators of the Socioeconomic Status–Obesity Association

Given that SES itself is difficult to change, one promising strategy for reducing socioeconomic inequities in obesity is to identify modifiable resilience factors that attenuate the effects of SES on obesity. However, relatively few studies have examined moderators of the association between SES and obesity. A 2023 systematic review examined the moderating role of built environmental features on the association between residence in a low-SES neighborhood and overweight or obesity [16]. The authors concluded that there was no evidence of moderation by street connectivity, population density, the food environment, access to physical activity facilities, or several perceived environmental attributes (eg, perceived neighborhood safety). Findings were mixed for the moderating role of walkability. Advancements in assessing built environmental features, such as satellite images, have become more widely available since many of these studies were published. Future research could continue to provide insights using more precise and comprehensive approaches to measuring the built environment. Additionally, many studies have shown that the association between SES and obesity differs by sex, with steeper gradients among females vs males [15, 16, 23]. While these findings do not provide a modifiable target for intervention, these sex differences can generate hypotheses about sex-specific moderators. For example, greater access to opportunities for physical activity among males vs females may help buffer the effects of low-SES on obesity [66].

## Discussion

In this paper, we summarized 19 review articles and meta-analyses about the association between SES and obesity published in the last two decades. While our review is nonexhaustive, we have summarized a range of study designs and highlighted key findings. We concluded that the patterns first identified in the 1980s have persisted worldwide, from childhood to adulthood, and across sexes, although the association tends to be stronger in females. In many, but not all cases, the findings are mostly robust regardless of which SES or obesity indicator was used [15, 54].

Opportunities for methodological advances include the need for prospective study designs in which SES, adiposity, and potential mediators and moderators are measured at multiple time points. This study design is critical for understanding the directionality of these associations by establishing temporality. Longitudinal designs that employ a life course perspective can reveal [1] life stages when actions to prevent obesity have the potential to be most effective [2], whether there are long-term effects of exposure to adverse socioeconomic conditions (eg, whether SES in early life can affect obesity in adulthood independent of adult SES), and [3] if a longer duration of exposure to adverse socioeconomic conditions is associated with a higher risk or greater severity of obesity [14, 64, 67]. One recent example is a 2022 study by Aris and colleagues [68] using data from the Environmental influences on Child Health Outcomes (ECHO) Consortium that showed neighborhood-level indices of socioeconomic adversity at birth were associated with a lower risk of obesity from childhood to adolescence . Studies that assess obesity outcomes at multiple time points are particularly valuable because growth trajectories have been linked to adverse cardiometabolic outcomes; and transitional periods from one life stage to another may be critical periods for the development of obesity [69, 70].

In addition to longitudinal studies that employ a life course perspective, research is needed with rigorous measures of body composition and body fat distribution that can overcome the limitations of BMI [27, 28]. Distinguishing between lean and fat mass, or between central and peripheral adiposity, using methods such as skinfold measurements and air displacement plethysmography, can enhance our understanding of the health effects of socioeconomic conditions and also provide insight into mechanistic pathways. Objective biomarkers of cardiovascular risk, such as serum lipids, and their association with SES also merit further study. Among the few studies that have been conducted, some show a positive association, whereas others show no association [71, 72]. The use of these types of finer-tuned measures of adiposity and metabolic health can inform the development of clinical and public health interventions through the identification of 1) individuals with central adiposity and a high body percentage whose health status may be masked by a normal BMI, and 2) metabolically healthy individuals with a BMI in the overweight range who may be a lower priority for some interventions [73].

Another opportunity for scientific innovation is to examine biological mediators of the SES-obesity association [61, 74]. In 2023, scientists at the National Institutes of Health highlighted specific biological pathways that merit further research. First, low SES may be a source of chronic stress, which activates the sympathetic nervous system and hypothalamic-pituitary-adrenal axis leading to higher levels of catecholamines and cortisol [61]. Yet, catecholamine resistance due to the stress of socioeconomic adversity, and its effect on fat accumulation over time, is not well understood. Second, little is known about the mediating role of adipokines in the SES-obesity association. Specifically, more observational studies can examine how the SDoH, including low SES, alter leptin resistance and the secretion and function of adiponectin [61]. These types of studies could build on previous work suggesting a mediating role for adipokines on the association between other environmental exposures (ie, air pollution) and risk factors for cardiovascular disease [75]. Another opportunity for scientific innovation is to identify novel environmental mediators and moderators of the SES-obesity association that are increasingly pervasive in some populations. Examples include the expanded use of social media and mobile technologies in developed economies, and increased exposure to highly processed foods, chemicals in household products, and air pollutants in developing economies due to globalization and industrialization [76-78]. Finally, many SDoH that are intertwined with SES have been linked to obesity. For example, neighborhood environments, including perceptions of safety and social cohesion, are correlated with socioeconomic status and obesity [79]. Another example is the association between the social constructs of self-identified race and ethnicity and obesity, which may be partially explained by structural racism, residential segregation, acculturation, or socioeconomic conditions [12, 80-83]. Future studies that measure multiple SDoH in the same population can help establish the independent effects of these factors on obesity. Many large-scale studies, such as the National Health and Nutrition Examination Survey (NHANES) and the National Institutes of Health ECHO Consortium are already collecting these types of data, and deidentified data sets are available for public download [84, 85]. Other opportunities for innovation include linking individual-level data on obesity-related health outcomes to publicly available data sources of upstream and midstream SDoH and related indices, many of which have been constructed by the US Census Bureau and the Centers for Disease Control and Prevention's PLACES database [86, 87]. Examples include the Structural Racism Effect Index, the Index of Concentration at the Extremes, the Social Vulnerability Index, the Child Opportunity Index, The Social Deprivation Index, the Area Deprivation Index, and the CDC's Environmental Justice Index. While many of these indices have been linked to health, their relationship with obesity and other dimensions of cardiovascular health are not well understood [68, 80, 88-97].

While intervention studies were not the focus of this review paper, we note that systematic reviews and meta-analyses published in 2023 and 2024 summarized targeted efforts to reduce obesity among youth and adults from low-SES households [98, 99]. Most intervention studies among individuals with low SES show small or nonsignificant decreases in BMI in the short term, or unsustained benefits in the long term [98, 99]. Examples of interventions that have been less effective among individuals with low SES include social support for weight loss and health education to encourage individual behavior change. In contrast, there is evidence that financial incentives, interactive feedback (ie, receiving real-time feedback on progress toward behavior change goals via mobile application or text message), tailored weight loss programs delivered by primary care providers, community-based strategies, and policies that include structural changes to the environment may be promising approaches [98-101]. In developing economies worldwide, obesity-prevention efforts are less common, likely due to the concentration of obesity among individuals with high SES, as well as the continuing need to prioritize efforts to reduce undernutrition [102]. While efforts to improve SES itself are not within the scope of public health, it should be noted that higher SES attenuates the association between higher genetic risk for obesity and obesity [103, 104].

We conclude that the steady worldwide increase in obesity (Supplemental Fig. 1) [36, 105] since the 1970s and the persistence of the socioeconomic gradient over decades reinforce the need for continued research on the association between SES and obesity [39]. Efforts to mitigate these inequities are incomplete, with few interventions demonstrating significant and sustained benefits for obesity prevention among socioeconomic groups with the highest obesity burden. Existing gaps in the literature, including understanding causal pathways and identifying factors that buffer the effects of socioeconomic conditions on obesity, provide novel opportunities for innovation.

## Data Availability

Data sharing is not applicable to this article as no data sets were generated or analyzed during this study.

## Abbreviations

BMI: body mass index
SDoH: social determinants of health
SES: socioeconomic status

## References

[1] O'Hearn M , Lauren BN, Wong JB, Kim DD, Mozaffarian D. Trends and disparities in cardiometabolic health among U.S. Adults, 1999-2018. J Am Coll Cardiol. 2022;80(2):138‐151. 35798448 10.1016/j.jacc.2022.04.046 PMC10475326

[2] Miranda JJ , Barrientos-Gutiérrez T, Corvalan C, et al Understanding the rise of cardiometabolic diseases in low- and middle-income countries. Nat Med. 2019;25(11):1667‐1679. 31700182 10.1038/s41591-019-0644-7

[3] Riazi K , Azhari H, Charette JH, et al The prevalence and incidence of NAFLD worldwide: a systematic review and meta-analysis. Lancet Gastroenterol Hepatol. 2022;7(9):851‐861. 35798021 10.1016/S2468-1253(22)00165-0

[4] Quek YH , Tam WWS, Zhang MWB, Ho RCM. Exploring the association between childhood and adolescent obesity and depression: a meta-analysis. Obes Rev. 2017;18(7):742‐754. 28401646 10.1111/obr.12535

[5] Corica F , Bianchi G, Corsonello A, Mazzella N, Lattanzio F, Marchesini G. Obesity in the context of aging: quality of life considerations. Pharmacoeconomics. 2015;33(7):655‐672. 25420750 10.1007/s40273-014-0237-8

[6] Weintraub WS . The economic burden of illness. JAMA Netw Open. 2023;6(3):e232663. 36912842 10.1001/jamanetworkopen.2023.2663

[7] Economic costs of diabetes in the U.S. in 2017. Diabetes Care. 2018;41(5):917‐928. 29567642 10.2337/dci18-0007 PMC5911784

[8] Centers for Disease Control and Prevention . *Health and Economic Costs of Chronic Diseases*. CDC; 2024. https://www.cdc.gov/chronicdisease/about/costs/index.htm

[9] Heslehurst N , Vieira R, Akhter Z, et al The association between maternal body mass index and child obesity: a systematic review and meta-analysis. PLoS Med. 2019;16(6):e1002817. 31185012 10.1371/journal.pmed.1002817 PMC6559702

[10] Zhang J , Clayton GL, Overvad K, Olsen A, Lawlor DA, Dahm CC. Body mass index in parents and their adult offspring: a systematic review and meta-analysis. Obes Rev. 2024;25(1):e13644. 37783229 10.1111/obr.13644 PMC10909538

[11] Gao C , Sanchez KM, Lovinsky-Desir S. Structural and social determinants of inequitable environmental exposures in the United States. Clin Chest Med. 2023;44(3):451‐467. 37517826 10.1016/j.ccm.2023.03.002

[12] Dougherty GB , Golden SH, Gross AL, Colantuoni E, Dean LT. Measuring structural racism and its association with BMI. Am J Prev Med. 2020;59(4):530‐537. 32863079 10.1016/j.amepre.2020.05.019 PMC8147662

[13] Marmot MG , Bell R. Action on health disparities in the United States: commission on social determinants of health. JAMA. 2009;301(11):1169‐1171. 19293419 10.1001/jama.2009.363

[14] Jones NL , Gilman SE, Cheng TL, Drury SS, Hill CV, Geronimus AT. Life course approaches to the causes of health disparities. Am J Public Health. 2019;109(S1):S48‐S55. 30699022 10.2105/AJPH.2018.304738 PMC6356123

[15] Sares-Jäske L , Grönqvist A, Mäki P, Tolonen H, Laatikainen T. Family socioeconomic status and childhood adiposity in Europe—a scoping review. Prev Med. 2022;160:107095. 35594926 10.1016/j.ypmed.2022.107095

[16] Selvakumaran S , Lin CY, Hadgraft N, Chandrabose M, Owen N, Sugiyama T. Area-level socioeconomic inequalities in overweight and obesity: systematic review on moderation by built-environment attributes. Health Place. 2023;83:103101. 37625238 10.1016/j.healthplace.2023.103101

[17] Morenz AM , Liao JM, Au DH, Hayes SA. Area-Level socioeconomic disadvantage and health care spending: a systematic review. JAMA Netw Open. 2024;7(2):e2356121. 38358740 10.1001/jamanetworkopen.2023.56121 PMC10870184

[18] Gautam N , Dessie G, Rahman MM, Khanam R. Socioeconomic status and health behavior in children and adolescents: a systematic literature review. Front Public Health. 2023;11:1228632. 37915814 10.3389/fpubh.2023.1228632 PMC10616829

[19] de Mestral C , Stringhini S. Socioeconomic status and cardiovascular disease: an update. Curr Cardiol Rep. 2017;19(11):115. 28965316 10.1007/s11886-017-0917-z

[20] Wang T , Li Y, Zheng X. Association of socioeconomic status with cardiovascular disease and cardiovascular risk factors: a systematic review and meta-analysis. Z Gesundh Wiss. 2023; doi:10.1007/s10389-023-01825-4 PMC9867543 36714072

[21] Chen E , Miller GE. Socioeconomic status and health: mediating and moderating factors. Annu Rev Clin Psychol. 2013;9(1):723‐749. 23245339 10.1146/annurev-clinpsy-050212-185634

[22] International Monetary Fund . *Income Inequality*. IMF; 2024. https://www.imf.org/en/Topics/Inequality/introduction-to-inequality

[23] Sobal J , Stunkard AJ. Socioeconomic status and obesity: a review of the literature. Psychol Bull. 1989;105(2):260‐275. 2648443 10.1037/0033-2909.105.2.260

[24] Jones-Smith JC , Gordon-Larsen P, Siddiqi A, Popkin BM. Cross-national comparisons of time trends in overweight inequality by socioeconomic status among women using repeated cross-sectional surveys from 37 developing countries, 1989-2007. Am J Epidemiol. 2011;173(6):667‐675. 21300855 10.1093/aje/kwq428 PMC3105263

[25] Subramanian SV , Perkins JM, Ozaltin E, Davey Smith G. Weight of nations: a socioeconomic analysis of women in low- to middle-income countries. Am J Clin Nutr. 2011;93(2):413‐421. 21068343 10.3945/ajcn.110.004820 PMC3021433

[26] Traore SS , Bo Y, Kou G, Lyu Q. Socioeconomic inequality in overweight/obesity among US children: NHANES 2001 to 2018. Front Pediatr. 2023;11:1082558. 36873636 10.3389/fped.2023.1082558 PMC9978798

[27] Bridger Staatz C , Kelly Y, Lacey RE, et al Life course socioeconomic position and body composition in adulthood: a systematic review and narrative synthesis. Int J Obes (Lond). 2021;45(11):2300‐2315. 34316000 10.1038/s41366-021-00898-z PMC8528709

[28] Bridger Staatz C , Kelly Y, Lacey RE, et al Socioeconomic position and body composition in childhood in high- and middle-income countries: a systematic review and narrative synthesis. Int J Obes. 2021;45(11):2316‐2334. 10.1038/s41366-021-00899-y PMC8528703 34315999

[29] Staatz CB , Kelly Y, Lacey RE, Hardy R. Area-level and family-level socioeconomic position and body composition trajectories: longitudinal analysis of the UK millennium cohort study. Lancet Public Health. 2021;6(8):e598‐e607. 34332672 10.1016/S2468-2667(21)00134-1 PMC8342403

[30] Martorell R , Khan LK, Hughes ML, Grummer-Strawn LM. Obesity in Latin American women and children. J Nutr. 1998;128(9):1464‐1473. 9732306 10.1093/jn/128.9.1464

[31] McLaren L . Socioeconomic status and obesity. Epidemiol Rev. 2007;29(1):29‐48. 17478442 10.1093/epirev/mxm001

[32] Himmelgreen DA , Cantor A, Arias S, Romero Daza N. Using a biocultural approach to examine migration/globalization, diet quality, and energy balance. Physiol Behav. 2014;134:76‐85. 24463063 10.1016/j.physbeh.2013.12.012

[33] Popkin BM , Reardon T. Obesity and the food system transformation in Latin America. Obes Rev. 2018;19(8):1028‐1064. 29691969 10.1111/obr.12694 PMC6103889

[34] Haghjoo P , Siri G, Soleimani E, Farhangi MA, Alesaeidi S. Screen time increases overweight and obesity risk among adolescents: a systematic review and dose-response meta-analysis. BMC Prim Care. 2022;23(1):161. 35761176 10.1186/s12875-022-01761-4 PMC9238177

[35] Porterfield F , Shapoval V, Langlet J, Samouda H, Stanford FC. Digital biometry as an obesity diagnosis tool: a review of current applications and future directions. Life (Basel). 2024;14(8):947. 39202689 10.3390/life14080947 PMC11355313

[36] Repository for Supplemental Material. Deposited on September 25, 2024. 10.6084/m9.figshare.27103861.

[37] American Psychological Association Task Force on Socioeconomic Status . Report of the APA Task Force on Socioeconomic Status. 2007.

[38] Oakes JM , Rossi PH. The measurement of SES in health research: current practice and steps toward a new approach. Soc Sci Med. 2003;56(4):769‐784. 12560010 10.1016/s0277-9536(02)00073-4

[39] World Health Organization . *Obesity and Overweight Fact Sheet*. WHO; 2024. http://www.who.int/mediacentre/factsheets/fs311/en/

[40] Lister NB , Baur LA, Felix JF, et al Child and adolescent obesity. Nat Rev Dis Primers. 2023;9(1):24. 37202378 10.1038/s41572-023-00435-4

[41] Prentice AM , Jebb SA. Beyond body mass index. Obes Rev. 2001;2(3):141‐147. 12120099 10.1046/j.1467-789x.2001.00031.x

[42] Garn SM . Family-line and socioeconomic factors in fatness and obesity. Nutr Rev. 1986;44(12):381‐386. 3822266 10.1111/j.1753-4887.1986.tb07581.x

[43] Deurenberg P . Universal cut-off BMI points for obesity are not appropriate. Br J Nutr. 2001;85(2):135‐136. 11280336 10.1079/bjn2000273

[44] Pi-Sunyer FX . The epidemiology of central fat distribution in relation to disease. Nutr Rev. 2004;62(7):S120‐S126. 15387477 10.1111/j.1753-4887.2004.tb00081.x

[45] Wang Y , Beydoun MA. The obesity epidemic in the United States–gender, age, socioeconomic, racial/ethnic, and geographic characteristics: a systematic review and meta-regression analysis. Epidemiol Rev. 2007;29(1):6‐28. 17510091 10.1093/epirev/mxm007

[46] Shrewsbury V , Wardle J. Socioeconomic status and adiposity in childhood: a systematic review of cross-sectional studies 1990-2005. Obesity (Silver Spring). 2008;16(2):275‐284. 18239633 10.1038/oby.2007.35

[47] Dinsa GD , Goryakin Y, Fumagalli E, Suhrcke M. Obesity and socioeconomic status in developing countries: a systematic review. Obes Rev. 2012;13(11):1067‐1079. 22764734 10.1111/j.1467-789X.2012.01017.x PMC3798095

[48] Cohen AK , Rai M, Rehkopf DH, Abrams B. Educational attainment and obesity: a systematic review. Obes Rev. 2013;14(12):989‐1005. 23889851 10.1111/obr.12062 PMC3902051

[49] Mohammed SH , Habtewold TD, Birhanu MM, et al Neighbourhood socioeconomic status and overweight/obesity: a systematic review and meta-analysis of epidemiological studies. BMJ Open. 2019;9(11):e028238. 10.1136/bmjopen-2018-028238 PMC6886990 31727643

[50] Buoncristiano M , Williams J, Simmonds P, et al Socioeconomic inequalities in overweight and obesity among 6- to 9-year-old children in 24 countries from the World Health Organization European region. Obes Rev. 2021;22(S6):e13213. 34184399 10.1111/obr.13213

[51] Kim TJ , von dem Knesebeck O. Income and obesity: what is the direction of the relationship? A systematic review and meta-analysis. BMJ Open. 2018;8(1):e019862. 10.1136/bmjopen-2017-019862 PMC5781054 29306894

[52] Newton S , Braithwaite D, Akinyemiju TF. Socio-economic status over the life course and obesity: systematic review and meta-analysis. PLoS One. 2017;12(5):e0177151. 28510579 10.1371/journal.pone.0177151 PMC5433719

[53] van den Berg G , van Eijsden M, Vrijkotte TG, Gemke RJ. BMI may underestimate the socioeconomic gradient in true obesity. Pediatr Obes. 2013;8(3):e37‐e40. 23283767 10.1111/j.2047-6310.2012.00133.x

[54] Witkam R , Gwinnutt JM, Humphreys J, Gandrup J, Cooper R, Verstappen SMM. Do associations between education and obesity vary depending on the measure of obesity used? A systematic literature review and meta-analysis. SSM Popul Health. 2021;15:100884. 34401462 10.1016/j.ssmph.2021.100884 PMC8350379

[55] Gebremariam MK , Lien N, Nianogo RA, Arah OA. Mediators of socioeconomic differences in adiposity among youth: a systematic review. Obes Rev. 2017;18(8):880‐898. 28434193 10.1111/obr.12547

[56] Mech P , Hooley M, Skouteris H, Williams J. Parent-related mechanisms underlying the social gradient of childhood overweight and obesity: a systematic review. Child Care Health Dev. 2016;42(5):603‐624. 27316858 10.1111/cch.12356

[57] Cronin FM , Hurley SM, Buckley T, et al Mediators of socioeconomic differences in overweight and obesity among youth in Ireland and the UK (2011-2021): a systematic review. BMC Public Health. 2022;22(1):1585. 35987999 10.1186/s12889-022-14004-z PMC9392918

[58] Claassen MA , Klein O, Bratanova B, Claes N, Corneille O. A systematic review of psychosocial explanations for the relationship between socioeconomic status and body mass index. Appetite. 2019;132:208‐221. 30217475 10.1016/j.appet.2018.07.017

[59] Loucks EB , Huang YT, Agha G, et al Epigenetic mediators between childhood socioeconomic disadvantage and mid-life body mass Index: the new England family study. Psychosom Med. 2016;78(9):1053‐1065. 27768648 10.1097/PSY.0000000000000411 PMC7380568

[60] Liu RS , Aiello AE, Mensah FK, et al Socioeconomic status in childhood and C reactive protein in adulthood: a systematic review and meta-analysis. J Epidemiol Community Health. 2017;71(8):817‐826. 28490476 10.1136/jech-2016-208646 PMC5843476

[61] Baez AS , Ortiz-Whittingham LR, Tarfa H, et al Social determinants of health, health disparities, and adiposity. Prog Cardiovasc Dis. 2023;78:17‐26. 37178992 10.1016/j.pcad.2023.04.011 PMC10330861

[62] Hui LL , Leung GM, Schooling CM. Social patterning in adiposity in adolescence: prospective observations from the Chinese birth cohort “children of 1997ˮ. PLoS One. 2016;11(1):e0146198. 26735134 10.1371/journal.pone.0146198 PMC4703380

[63] Keleher MR , Zaidi R, Shah S, et al Maternal high-fat diet associated with altered gene expression, DNA methylation, and obesity risk in mouse offspring. PLoS One. 2018;13(2):e0192606. 29447215 10.1371/journal.pone.0192606 PMC5813940

[64] Ben-Shlomo Y , Kuh D. A life course approach to chronic disease epidemiology: conceptual models, empirical challenges and interdisciplinary perspectives. Int J Epidemiol. 2002;31(2):285‐293. 11980781

[65] Hewagalamulage SD , Lee TK, Clarke IB, Henry BA. Stress, cortisol, and obesity: a role for cortisol responsiveness in identifying individuals prone to obesity. Domest Anim Endocrinol. 2016;56:S112‐S120. 27345309 10.1016/j.domaniend.2016.03.004

[66] Trumpeter NN , Wilson DK. Positive action for Today's health (PATH): sex differences in walking and perceptions of the physical and social environment. Environ Behav. 2014;46(6):745‐767. 26740707 10.1177/0013916513480860 PMC4698897

[67] Baird J , Jacob C, Barker M, et al Developmental origins of health and disease: a lifecourse approach to the prevention of non-communicable diseases. Healthcare (Basel). 2017;5(1):14. 28282852 10.3390/healthcare5010014 PMC5371920

[68] Aris IM , Perng W, Dabelea D, et al Associations of neighborhood opportunity and social vulnerability with trajectories of childhood body mass Index and obesity among US children. JAMA Netw Open. 2022;5(12):e2247957. 36547983 10.1001/jamanetworkopen.2022.47957 PMC9857328

[69] Dietz WH . Critical periods in childhood for the development of obesity. Am J Clin Nutr. 1994;59(5):955‐959. 8172099 10.1093/ajcn/59.5.955

[70] Peneau S , Giudici KV, Gusto G, et al Growth trajectories of body mass Index during childhood: associated factors and health outcome at adulthood. J Pediatr. 2017;186:64‐71.e61. 28283258 10.1016/j.jpeds.2017.02.010

[71] Espírito Santo LR , Faria TO, Silva CSO, et al Socioeconomic status and education level are associated with dyslipidemia in adults not taking lipid-lowering medication: a population-based study. Int Health. 2022;14(4):346‐353. 31693111 10.1093/inthealth/ihz089 PMC10575599

[72] Odutayo A , Gill P, Shepherd S, et al Income disparities in absolute cardiovascular risk and cardiovascular risk factors in the United States, 1999-2014. JAMA Cardiol. 2017;2(7):782‐790. 28593301 10.1001/jamacardio.2017.1658 PMC5710615

[73] Blüher M . Metabolically healthy obesity. Endocr Rev. 2020;41(3):bnaa004. 32128581 10.1210/endrev/bnaa004 PMC7098708

[74] Krueger PM , Reither EN. Mind the gap: race/ethnic and socioeconomic disparities in obesity. Curr Diab Rep. 2015;15(11):95. 26377742 10.1007/s11892-015-0666-6 PMC4947380

[75] Wolf K , Popp A, Schneider A, et al Association between long-term exposure to air pollution and biomarkers related to insulin resistance, subclinical inflammation, and adipokines. Diabetes. 2016;65(11):3314‐3326. 27605624 10.2337/db15-1567

[76] Oduro MS , Katey D, Morgan AK, Peprah P. Overweight/obesity is associated with problematic social media use: addressing problematic social media use could help reduce overweight/obesity among adolescents. Pediatr Obes. 2024;19(2):e13093. 38146210 10.1111/ijpo.13093

[77] Popkin BM , Ng SW. The nutrition transition to a stage of high obesity and noncommunicable disease prevalence dominated by ultra-processed foods is not inevitable. Obes Rev. 2022;23(1):e13366. 34632692 10.1111/obr.13366 PMC8639733

[78] Heindel JJ , Lustig RH, Howard S, Corkey BE. Obesogens: a unifying theory for the global rise in obesity. Int J Obes (Lond). 2024;48(4):449‐460. 38212644 10.1038/s41366-024-01460-3 PMC10978495

[79] Daniels KM , Schinasi LH, Auchincloss AH, Forrest CB, Diez Roux AV. The built and social neighborhood environment and child obesity: a systematic review of longitudinal studies. Prev Med. 2021;153:106790. 34506813 10.1016/j.ypmed.2021.106790

[80] Dyer Z , Alcusky MJ, Galea S, Ash A. Measuring the enduring imprint of structural racism on American neighborhoods. Health Aff (Millwood). 2023;42(10):1374‐1382. 37782878 10.1377/hlthaff.2023.00659 PMC10804769

[81] Mackey ER , Burton ET, Cadieux A, et al Addressing structural racism is critical for ameliorating the childhood obesity epidemic in black youth. Child Obes. 2022;18(2):75‐83. 34491828 10.1089/chi.2021.0153

[82] Andrea SB , Hooker ER, Messer LC, Tandy T, Boone-Heinonen J. Does the association between early life growth and later obesity differ by race/ethnicity or socioeconomic status? A systematic review. Ann Epidemiol. 2017;27(9):583‐592.e585. 28911983 10.1016/j.annepidem.2017.08.019 PMC6688753

[83] Skinner AC , Ravanbakht SN, Skelton JA, Perrin EM, Armstrong SC. Prevalence of obesity and severe obesity in US children, 1999-2016. Pediatrics. 2018;141(3):e20173459. 29483202 10.1542/peds.2017-3459 PMC6109602

[84] Eunice Kennedy Shriver National Insitute of Child Health and Human Development . *Data and Biospecimen Hub (DASH)*. Eunice Kennedy Shriver National Insitute of Child Health and Human Development; 2022. https://dash.nichd.nih.gov/

[85] Centers for Disease Control and Prevention National Center for Health Statistics (NCHS) . *About the National Health and Nutrition Examination Survey*. CDC; 2019. https://www.cdc.gov/nchs/nhanes/about_nhanes.htm

[86] U.S. Census Bureau . *American Community Survey (ACS)*. U.S. Census Bureau; 2023. https://www.census.gov/programs-surveys/acs

[87] Centers for Disease Control and Prevention . *Place and Health—CDC/ATSDR Social Vulnerability Index (CDC/ATSDR SVI): Overview*. CDC; 2024. https://www.atsdr.cdc.gov/placeandhealth/svi/index.html

[88] Kunin-Batson A , Carr C, Tate A, et al Interpersonal discrimination, neighborhood inequities, and Children's body mass Index: a descriptive, cross-sectional analysis. Fam Community Health. 2023;46(S1):S30‐s40. 37696014 10.1097/FCH.0000000000000372 PMC10503111

[89] Heneghan JA , Goodman DM, Ramgopal S. Hospitalizations at United States Children's hospitals and severity of illness by neighborhood child opportunity Index. J Pediatr. 2023;254:83‐90.e88. 36270394 10.1016/j.jpeds.2022.10.018

[90] Tyris J , Gourishankar A, Kachroo N, Teach SJ, Parikh K. The child opportunity Index and asthma morbidity among children younger than 5 years old in Washington, DC. J Allergy Clin Immunol. 2024;153(1):103‐110.e105. 37877904 10.1016/j.jaci.2023.08.034

[91] Aris IM , Rifas-Shiman SL, Jimenez MP, et al Neighborhood child opportunity Index and adolescent cardiometabolic risk. Pediatrics. 2021;147(2):e2020018903. 33479165 10.1542/peds.2020-018903 PMC7906069

[92] Wild LE , Walters M, Powell A, James KA, Corlin L, Alderete TL. County-level social vulnerability is positively associated with cardiometabolic disease in Colorado. Int J Environ Res Public Health. 2022;19(4):2202. 35206386 10.3390/ijerph19042202 PMC8872484

[93] Kurani SS , Heien HC, Sangaralingham LR, et al Association of area-level socioeconomic deprivation with hypoglycemic and hyperglycemic crises in US adults with diabetes. JAMA Netw Open. 2022;5(1):e2143597. 35040969 10.1001/jamanetworkopen.2021.43597 PMC8767428

[94] Giammarino AM , Qiu H, Bulsara K, et al Community socioeconomic deprivation predicts nonalcoholic steatohepatitis. Hepatol Commun. 2022;6(3):550‐560. 34668658 10.1002/hep4.1831 PMC8870017

[95] Sheets LR , Henderson Kelley LE, Scheitler-Ring K, et al An index of geospatial disadvantage predicts both obesity and unmeasured body weight. Prev Med Rep. 2020;18:101067. 32154094 10.1016/j.pmedr.2020.101067 PMC7056721

[96] Patel VR , Jella T, Gupta A, Nassel A, Ibrahim A, Hussaini SMQ. Association of neighborhood-level environmental injustice with health Status in the US. JAMA Intern Med. 2023;183(10):1162‐1163. 37578753 10.1001/jamainternmed.2023.2835 PMC10425860

[97] Martenies SE , Zhang M, Corrigan AE, et al Developing a national-scale exposure index for combined environmental hazards and social stressors and applications to the environmental influences on child health outcomes (ECHO) cohort. Int J Environ Res Public Health. 2023;20(14):6339. 37510572 10.3390/ijerph20146339 PMC10379099

[98] Harakeh Z , Preuhs K, Eekhout I, Lanting C, Klein Velderman M, van Empelen P. Behavior change techniques that prevent or decrease obesity in youth with a low socioeconomic Status: a systematic review and meta-analysis. Child Obes. 2023;20(2): 128‐140. 37204322 10.1089/chi.2022.0172

[99] Li P , Huang Y, Wong A. Behavioural strategies to reduce obesity among lower socio-economic adults living in high-income countries: a grades of recommendation, assessment, development and evaluation-assessed systematic review and meta-analysis of randomised controlled trials. Br J Nutr. 2024;131(3):544‐552. 37622175 10.1017/S0007114523001940

[100] Hillier-Brown FC , Bambra CL, Cairns JM, Kasim A, Moore HJ, Summerbell CD. A systematic review of the effectiveness of individual, community and societal-level interventions at reducing socio-economic inequalities in obesity among adults. Int J Obes (Lond). 2014;38(12):1483‐1490. 24813369 10.1038/ijo.2014.75 PMC4262962

[101] Beauchamp A , Backholer K, Magliano D, Peeters A. The effect of obesity prevention interventions according to socioeconomic position: a systematic review. Obes Rev. 2014;15(7):541‐554. 24629126 10.1111/obr.12161

[102] Popkin BM , Corvalan C, Grummer-Strawn LM. Dynamics of the double burden of malnutrition and the changing nutrition reality. Lancet. 2020;395(10217):65‐74. 31852602 10.1016/S0140-6736(19)32497-3 PMC7179702

[103] Cromer SJ , Lakhani CM, Mercader JM, et al Association and interaction of genetics and area-level socioeconomic factors on the prevalence of type 2 diabetes and obesity. Diabetes Care. 2023;46(5):944‐952. 36787958 10.2337/dc22-1954 PMC10154653

[104] Tommerup K , Ajnakina O, Steptoe A. Genetic propensity for obesity, socioeconomic position, and trajectories of body mass index in older adults. Sci Rep. 2021;11(1):20276. 34645866 10.1038/s41598-021-99332-7 PMC8514538

[105] World Health Organization . *The Global Health Observatory: Prevalence of Obesity among Adults, BMI ≥30 (Age-Standardized Estimate) (%)*. WHO; 2024. https://www.who.int/data/gho/data/indicators/indicator-details/GHO/prevalence-of-obesity-among-adults-bmi-=-30-(age-standardized-estimate)-(-)

